# Enhancement of the In Vitro Antitumor Effects of Berberine Chloride When Encapsulated within Small Extracellular Vesicles

**DOI:** 10.3390/pharmaceutics14091913

**Published:** 2022-09-09

**Authors:** Abir Salek, Mouna Selmi, Mahassen Barboura, M. Carmen Martinez, Leila Chekir-Ghedira, Ramaroson Andriantsitohaina

**Affiliations:** 1 PhyMedExp, University of Montpellier, INSERM, CNRS, 34295 Montpellier, France; 2 Research Unit Bioactive Natural Products and Biotechnology UR17ES49, Faculty of Dental Medicine of Monastir, University of Monastir, Monastir 5000, Tunisia

**Keywords:** antitumor efficacies, berberine hydrochloride, small extracellular vesicles

## Abstract

Berberine hydrochloride (BRB) is an isoquinoline alkaloid with promising anticancer efficacies. However, application of BRB had been hampered by its poor aqueous solubility, low gastrointestinal absorption, and rapid metabolism. The present study takes advantage of small extracellular vesicles (sEVs) to increase both stability and efficacy of BRB. sEVs from immature dendritic cells were produced and loaded with BRB. Proliferation, migration and Matrigel assay were performed, cycle arrest and nitric oxide (NO) production were evaluated in human breast cancer cell line (MDA-MB-231) and human umbilical vein endothelial cells (HUVECs). sEVs loaded with BRB formed a stable and homogenous population with a drug entrapment efficiency near to 42%. BRB loaded into sEVs was more potent than free BRB for MDA-MB-231 and endothelial proliferation, migration, and capillary-like formation in HUVECs. The mechanisms involved a blockade of cell cycle in G0/G1 phase, increased S phase and decreased of G2/M in MDA-MB-231 and HUVECs, and inhibition of NO production in HUVECs. Altogether, sEV-loaded BRB displayed higher effects than free BRB on different steps leading to its antitumor activity and anti-angiogenic properties in vitro. Thus, sEV formulation may be considered as an innovative approach and promising delivery of BRB to prevent tumorigenesis and angiogenesis.

## 1. Introduction

Breast cancer is the most common type of cancer among women worldwide [1]. Despite advances in the early detection and treatment of breast cancer, its mortality rate is still increasing each year. Currently, mammography, ultrasound, hormonotherapy, chemotherapy, and radiotherapy are the most efficient strategies recommended by different clinical guidelines for breast cancer diagnostic and treatment. Unfortunately, their sensitivity and specificity are not consistent among different studies [2]. Chemotherapy and radiotherapy efficiencies are limited by serious side effects, such as toxicity and by the development of drug resistance, especially in triple-negative breast cancer (TNBC). TNBC is a very aggressive subtype of breast cancer with less than 30% of five-year survival rate in case of metastasis. Thus, pharmacological-driven strategies exhibiting beneficial outcomes and safety approaches for the treatment of TNBC are needed [3]. Many natural compounds found in diets, termed phytochemicals, are now used to treat or prevent breast cancer. In the present study, Berberine hydrochloride (BRB) therapeutic properties have been evaluated. BRB is a naturally isoquinoline alkaloid, isolated from various Chinese medicinal plants such as Berberis vulgaris (barberry), Berberis aquifolium (Oregon grape), Berberis aristata (tree turmeric), and Tinospora cordifolia [4]. BRB showed great pharmacological interest due to its pleiotropic effects such antibacterial [5], antidiabetic [6], anti-inflammatory [7], and anticancer activities [8,9]. However, BRB is poorly soluble in an aqueous environment, and its modest bioavailability and stability, due to its low gastro-intestinal absorption and rapid metabolism reduce its effect both in vitro and in vivo. The main strategies to overcome these limitations are the use of nanoparticle formulations to enhance bioavailability and efficacy of small molecular compounds, such as BRB [10]. Among the different nanomedicine approaches, extracellular vesicles (EVs) are suitable candidate for drug delivery. Indeed, small EVs (sEVs), nano-scaled vesicles released by most cell types and involved in many physiological and pathophysiological processes possess the ability to overcome the above-mentioned bioavailability limitations. Among the characteristics of sEVs, they show reduced host immune responses following in vivo administration and improve stability of the cargo [11]. For instance, polyphenol loaded into sEVs showed higher effects on their target cells when compared to free polyphenols [12]. Indeed, sEVs released from cells treated with curcumin are enriched with miR-21 and induce a decrease in tumor cell growth and angiogenesis, and modulate the endothelial barrier organization [12]. We recently reported that the polyphenol delphinidin can be encapsulated into sEVs to target diseases associated with excessive angiogenesis including cancer [13]. In the present study, we propose sEVs as biological carriers to enhance both stability and efficacy of BRB, and therefore its anti-tumoral and anti-angiogenic pharmacological properties.

## 2. Materials and Methods

### 2.1. Cell Culture

The murine dendritic cell line, JAWS II, was obtained from the American Type Culture Collection (ATCC, Manassas, VA, USA). JAWS II was cultured in the presence of alpha-minimum essential media (Lonza, Basel, Switzerland) complemented with 20% fetal bovine serum (FBS, Life Technologies; Grand Island, NY, USA), 1% penicillin-streptomycin (Sigma-Aldrich, St. Quentin Fallavier, France), 4 mM L-glutamine (Lonza), 1 mM sodium pyruvate (Lonza), and 5 ng/mL of Granulocyte-Macrophage Colony Stimulating Factor (Miltenyi Biotec; San Diego, CA, USA). The cells were maintained in culture in a humified incubator at 37 °C and 5% CO_2_.

The MDA-MB-231 human breast cancer cells were purchased from ATCC and cultured at 5% CO_2_ at 37 °C in Dulbecco’s modified Eagle media (Lonza), supplemented with 10% FBS, 2 mM glutamine, 10% penicillin-streptomycin.

Human umbilical vein endothelial cells (HUVECs) were purchased from Lonza and grown at 37 °C and 5% CO_2_ in endothelial basal cell growth medium, supplemented with 2% FBS, 1% streptomycin-penicillin, 0.1% ascorbic acid, 0.1% human epidermal growth factor, 0.1% heparin, 0.1% VEGF, 0.1% gentamicin-amphotericin B, 0.04% hydrocortisone, 0.4% human bFGF, and 0.1% R3-IGF-1 (all from Lonza).

### 2.2. sEV Isolation

JAWS II cells were seeded at a density of 5 × 10^6^ cells/175 cm^2^ in complete growth medium and they were deprived in FBS before sEV isolation. Supernatants were subjected to serial centrifugations (300× *g* and then 2000× *g* for 10 min to eliminate cells and debris, respectively). Then, the supernatant was centrifuged at 20,000× *g* for 30 min to remove large EVs, and the resultant supernatant was recentrifuged at 200,000× *g* (Optima MAX-XP ultracentrifuge, MLA-50 rotor, Beckman Coulter, Villepinte, France) for 2 h to pellet sEVs. Finally, the pellet was washed with PBS recentrifuged at 200,000× *g* for 2 h and resuspended in 1 mL of PBS. sEVs were stored at −80 °C until further analysis. The amount of proteins associated with sEVs was quantified by Lowry method, using bovine serum albumin as reference.

### 2.3. Berberine Chloride (BRB) Loading and Entrapment Efficiency

BRB (Alfa Aesar, Thermofisher, Germany) was loaded into sEVs with a simple impregnation method by suspending BRB powder (2 mg/mL) with sEV samples (250 µg/mL) in 1 mL absolute ethanol for 2 h. The mixture was vortexed, sonicated for 5 min and incubated at 37 °C for 2 h under magnetic stirring. Then, it was subjected to ultracentrifugation for 2 h at 200,000× *g* at 37 °C. sEVs loaded with BRB can be distinguished as a yellowish band in the pellet. BRB-loaded sEVs were collected, washed and resuspended in PBS and stored at −80 °C until use. The amount of BRB loaded and its entrapment efficiency (EE) within sEVs were quantified using a spectrophotometer (UV-2600, SHIMADZU, Kyoto, Japan). BRB absorbance was measured at 346 nm and standard curve with different concentrations (10–100 µg/mL) of free BRB was performed. The percentages of BRB EE and loading were calculated according to the following Equations:

% EE = [(BRB added − untrapped BRB)/BRB added] × 100

% BRB loading = [mass of BRB in sEV/mass of sEV] × 100

### 2.4. Nanoparticle Tracking Analysis (NTA)

Native sEVs and sEVs loaded with BRB were suspended in 0.9% NaCl and filtered (0.2 µm); then loaded into the sample chamber of NanoSight NS 300 (Malvern Instruments, Worcestershire, UK) to determine size distribution as previously described [13]. Briefly, 60 s videos were recorded, and data analysis was performed with NTA software using the Stokes–Enstein Equation.

### 2.5. Dynamic Light Scattering

Size distribution, polydispersity index (PDI), and zeta potential of sEVs and sEV-loaded BRB samples were quantified through dynamic light scattering on a Zetasizer^®^ Nanoseries DTS 1060 (Malvern Instruments). Formulations were diluted in 0.9% NaCl and filtered (0.2 µm). Results were analyzed as a number weighted distribution curve for the evaluation of the size of sEVs.

### 2.6. Western Blotting Analysis

Native sEVs and sEVs loaded with BRB were separated on 4–12% polyacrylamide gel electrophoresis. Then, proteins were transferred to nitrocellulose membranes and incubated with the following antibodies: Alix, TSG101, CD63 (Santa Cruz Biotechnology, Santa Cruz, CA, USA), and ß-Actin (Sigma-Aldrich). Each membrane was then incubated with the corresponding secondary antibody: anti-mouse or anti-rabbit (Santa Cruz), and visualized with the Chemi-Smart, 5000 Imaging System using Chemi-capt software (Vilber Lourmat, Marne-la-Vallée, France).

### 2.7. Proliferation Assay

Cell numbers were evaluated with the Proliferation Assay kit CyQUANT (Invitrogen, Carlsbad, CA, USA). Briefly, 5 × 10^3^ MDA-MB-231 and 10^4^ HUVECs were seeded in a 96-well plate and allowed to attach for 24 h. Cells were treated with increasing concentrations (1.25 to 100 µg/mL) of BRB and sEV-loaded BRB for three different times (24, 48 and 72 h). Concentration of BRB within sEVs was calculated taking in consideration the %EE of BRB. Then, the medium was removed and the dye-binding solution was added. Cells were incubated at 37 °C for 30 min. The fluorescence levels were read on a fluorescent microplate reader (CLARIOstar^®^, BMG LABTECH, Ortenberg, Germany) with 485 nm excitation and 530 nm emissions filters. For the subsequent experiments, BRB and sEV-loaded BRB were used at the respective IC_50_ obtained for each cell type, being 20 µg/mL for free BRB and 5 µg/mL for sEV-loaded BRB for MDA-MB-231, and 40 µg/mL for BRB and 25 µg/mL for sEV-loaded BRB for HUVECs.

### 2.8. Cell Cycle Analysis

Cells were seeded at a density of 2 × 10^5^ (MDA-MB-231) and 3 × 10^5^ (HUVECs) cells/well in 6-well plates, respectively, and allowed to attach for 24 h. MDA-MB-231 were treated with 10 µg/mL of native sEVs, 20 µg/mL of BRB, or 5 µg/mL of sEV-loaded BRB, whereas HUVECs were treated with 10 µg/mL of native sEVs, 40 µg/mL of BRB, or 25 µg/mL of sEV-loaded BRB. After 24 h, cells were washed in PBS, before adding 0.2 mL of nuclear isolation medium 0.6% NP40, 50 µg/mL propidium iodide, 100 µg/mL RNase in PBS (all from Sigma Aldrich). Cells were then incubated in the dark for 60 min and analyzed with FC 500 MPL flow cytometer (Beckman Coulter).

### 2.9. Wound Healing Assay

Cells were seeded at 2 × 10^6^ cells/well in 12-well plates. When the cells reached about 90% of confluence, they were scratched with a sterile pipette tip, and the floating cells and debris were removed by washing with complete medium. MDA-MB-231 were then treated with 10 µg/mL of native sEVs, 20 µg/mL of BRB, and 5 µg/mL of sEV-loaded BRB, while HUVECs were treated with 10 µg/mL of native sEVs, 40 µg/mL of BRB, and 25 µg/mL of sEV-loaded BRB. Images of the plates were taken at T = 0 (immediately after healing), at T = 24 h, and at T = 48 h. The area of the scratch was quantified using Image J software. The cell migration was quantified as percentage of the surface recovery.

% of recovery = [(AT0 − AT24/48)/AT0] × 100

### 2.10. In Vitro Capillary Network Formation

HUVECs were seeded at 25 × 10^3^ cells/well in 15 µ-slide angiogenesis plate (Ibidi, Gräfelfing, Germany) precoated with Matrigel^®^ (Sigma-Aldrich). Briefly, 10 µL of Matrigel^®^ was added into a 15-well plate and allowed to solidify for 45 min at 37 °C. Then, cells were incubated with medium containing 10% FBS and allowed to adhere for 45 min, after which different treatments (phorbol myristate acetate as a positive control (20 ng/mL), native sEVs (10 µg/mL), BRB (40 µg/mL), and sEV-loaded BRB (25 µg/mL) were added for 24 h. Tube formation was observed with an optical microscope (Olympus CK40, Rungis, France). Quantification was performed by measuring the number of capillary-like structures using Image J software 1.46r (National Institutes of Health, Bethesda, MD, USA).

### 2.11. Nitric Oxide (NO) Production Assay

HUVECs were seeded at 3 × 10^4^ cells/well in 8 µ-slide plates (Ibidi). At 70–80% of confluence, cells were stimulated for 24 h and 48 h with 10 µg/mL of native sEVs, 40 µg/mL of BRB, and 25 µg/mL of sEV-loaded BRB. After 24 h or 48 h, cells were washed with PBS and incubated with a fluorescent NO indicator 4,5-diaminofuorescein diacetate (5 μM; Santa Cruz Biotechnology) at 37 °C for 30 min. Then, cells were washed twice with cold PBS and fixed in paraformaldehyde at 4% for 20 min at room temperature in the dark. Cells were then washed with PBS and analyzed by confocal microscopy. From each well, a minimum of 4 randomly selected images were captured with a LSM 700 confocal microscope (Carl Zeiss, Jena, Germany).

### 2.12. Statistical Analysis

Data are expressed as mean values ± SEM. The significance of the differences between groups was determined by ANOVA, followed by Tukey’s or Sidak’s multiple comparisons test. *p* Values < 0.05 were considered significant.

## 3. Results

### 3.1. sEV Characterization, BRB Loading, and Entrapment Efficiency

**Figure 1:**
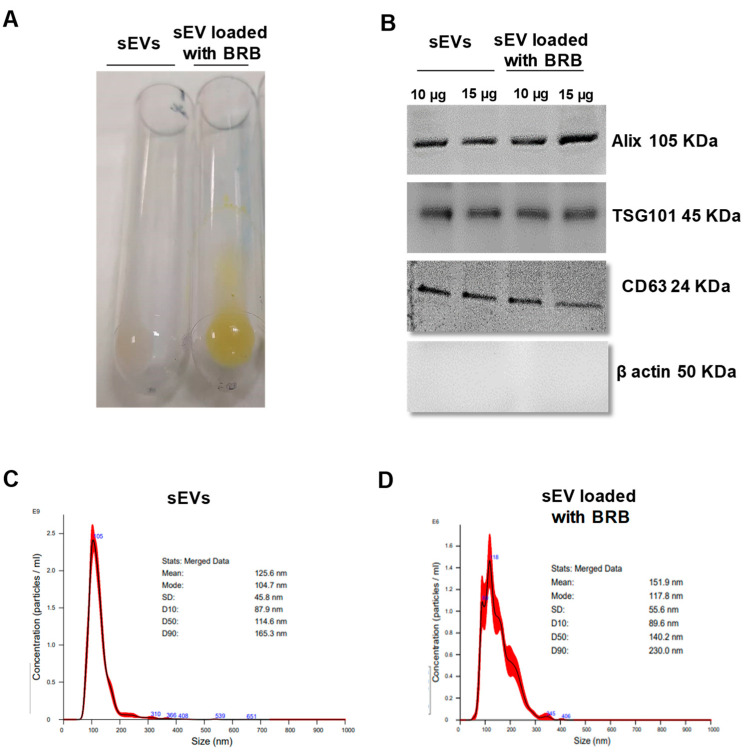
(**A**) Incorporation of berberine (BRB 2 mg/mL) into small extracellular vesicles (sEVs 250 µg/mL). Representative picture of sEVs loaded with BRB and native sEVs; sEVs loaded with BRB are distinguished as a yellowish band in the pellet. (**B**) Western blot analysis of Alix, TSG101, CD63, and β actin expression in sEVs and sEVs loaded with BRB. Size distributions of native sEVs (**C**) and sEV loaded with BRB (**D**) based on NTA measurements.

To assess the feasibility of using JAWS II-derived sEVs as drug vehicles, the entrapment efficiency (EE%) of BRB within sEVs was calculated by determining the amount of remaining free BRB. BRB was indeed loaded into sEVs, as shown by the yellow colored of the loaded sEVs with BRB (Figure 1A). Table 1 showed that the EE% of the formulation of sEVs loaded with BRB was 41.6 ± 1.7%, implying that BRB was effectively loaded into the sEVs. Also, both types of sEVs, native and those loaded with BRB, expressed sEV markers, such as Alix, TSG101 and CD63 at similar level, whereas they did not express ß-actin, a marker of large EVs (Figure 1B). The particle size, PDI, and zeta potential of native sEVs and sEVs loaded with BRB were determined by NTA and Zetasizer. The results (Table 1) showed that the mean particle size determined by NTA (Figure 1C,D) of sEVs loaded with BRB was (109.6 ± 11.5 nm) similar to the size of the native sEVs (101.13 ± 16.02 nm). Thus, the entrapment of free BRB into sEVs did not significantly modify the particle size. In addition, native sEVs and sEVs loaded with BRB formed a stable polydispersed suspension with a PDI value below 0.3. Besides, the zeta potential of sEVs loaded with BRB and native sEVs ranged from −5.4 ± 13 mV and −11.3 ± 8.4 mV, respectively, indicating a stable nanoparticule suspension. Taken together, these data demonstrated that sEVs are possible BRB carriers with homogeneous particle size and steady zeta potential.

### 3.2. Effects of Free BRB and sEV-Loaded BRB on Cell Proliferation

**Figure 2:**
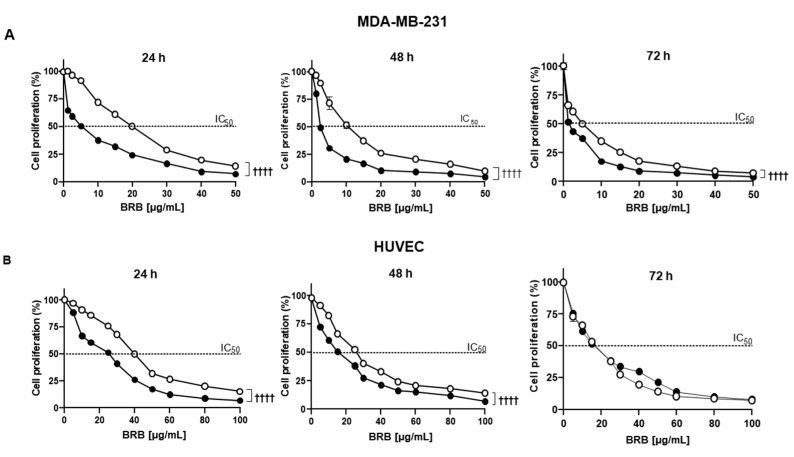
Cell proliferation assay. BRB (white circles) and sEV-loaded BRB (black circles) decreased cell proliferation of MDA-MB-231 (**A**) and HUVEC cells (**B**) after 24 h, 48 h, and 72 h of treatment. Data are expressed as mean ± SEM of three independent experiments. **^††††^** *p* < 0.0001.

In both MDA-MB-231 and HUVECs, native sEVs did not significantly modify cell proliferation up to 10 µg/mL at any time tested (not shown). At higher concentrations, sEVs induced a slight reduction of cell proliferation. In MDA-MB-231, free BRB inhibited cell proliferation in a concentration- and time-dependent manner with a maximal effect reached at 40 µg/mL after 24 h, 48 h, and 72 h of treatment (Figure 2A). In the same manner, sEV-loaded BRB inhibited cell proliferation in a concentration- and time-dependent manner. Interestingly, sEV-loaded BRB displayed higher efficacy in reducing cell proliferation compared to BRB alone. Indeed, the IC_50_ of sEV-loaded BRB was 4 times lower than the one obtained with free BRB at any time studied (Table 2).

In HUVECs, free BRB inhibited cell proliferation in a concentration and time-dependent manner with a maximal effect reached at 80 µg/mL after 24 h, and at 60 µg/mL after 48 h and 72 h of treatment (Figure 2B). The efficacy of sEV-loaded BRB was significantly greater than free BRB at any concentration after 24 h and 48 h (Figure 2B). No further efficacy was obtained at 72 h. The IC_50_ of sEV-loaded BRB was significantly lower than free BRB (about 2 times) at 24 h and 48 h. These results suggest that when loaded into sEVs, BRB is more potent than free BRB in decreasing cell proliferation.

According to the previous results, for the following experiments, free BRB and sEV-loaded BRB were used at their respective IC_50_ for each cell type.

### 3.3. Effects of Free BRB and sEV-Loaded BRB on Cell Cycle Progression

The effects of sEVs, free BRB, and sEV-loaded BRB on MDA-MB-231 cells and HUVECs on cell cycle distribution were evaluated. sEVs alone did not modify cell cycle on both cell types. At 24 h, free BRB (20 and 40 μg/mL for MDA-MB-231 cells and HUVECs, respectively) and sEV-loaded BRB (5 or 25 μg/mL for MDA-MB-231 cells and HUVECs, respectively) induced a diminution of G_0_/G_1_ phase associated with an increased in S phase in both cell types. This effect was associated with a decrease in G_2_/M phase in the two cell types although it was not significant in MDA-MB-231 (Supplementary Materials Figure S1). We conclude that BRB and sEV-loaded BRB arrested the cells at S phase, and thereby prevented cell cycle progression to G2/M phase.

### 3.4. Effects of Free BRB and sEV-Loaded BRB on Cell Migration

**Figure 3:**
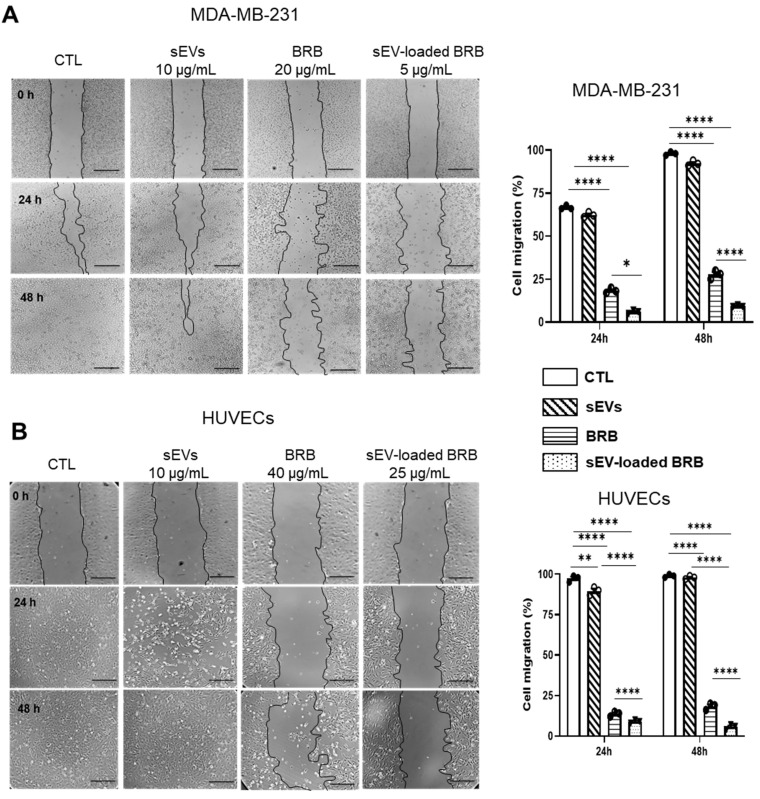
Wound healing assay of MDA-MB-231 (**A**) and HUVECs (**B**) after sEVs, BRB, and sEV-loaded BRB treatment. Cell migration was measured at T = 0 h, T = 24 h, and T = 48 h and analyzed as described in Methods. Horizontal bar = 200 µm. Data are expressed as mean ± SEM of 3–4 independent experiments. * *p* < 0.05, ** *p* < 0.01, **** *p* < 0.0001.

As shown on Figure 3, sEVs alone had no effect on cell migration while the treatment with free BRB (20 and 40 μg/mL for MDA-MB-231 and HUVECs, respectively) and sEV-loaded BRB (5 and 25 μg/mL for MDA-MB-231 and HUVECs, respectively) induced a significant decrease in cell migration, compared to the control and native sEV groups in which the scratch was completely closed after 48 h. Interestingly, the inhibitory effect of sEV-loaded BRB was significantly greater than free BRB in both cell types at any time tested.

### 3.5. Effects of Free BRB and sEV-Loaded BRB on Angiogenesis

**Figure 4:**
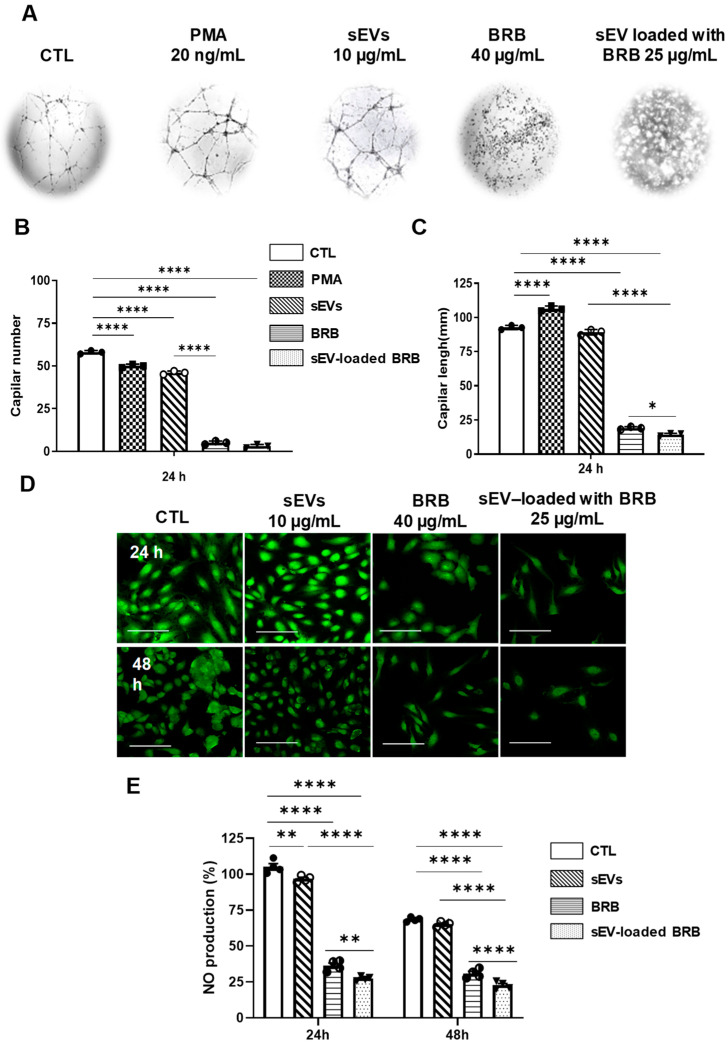
In vitro angiogenesis and NO production assays. (**A**) Representative phase-contrast micrographs of capillary structures in cultured HUVECs exposed 24 h to PMA, sEVs, BRB, and sEV-loaded BRB. Quantification of capillary number (**B**) and length (**C**). (**D**) Representative confocal images showing the decrease on NO production induced by 24 h and 48 h treatment with BRB and sEV-loaded BRB. Images were quantified using ImageJ (**E**). Scale bar = 50 µm. Data were shown as mean + SEM of 3–4 independent experiments. * *p* < 0.05, ** *p* < 0.01, **** *p* < 0.0001.

In the absence of treatment, HUVECs restructured and formed capillary-like structures (Figure 4A). Phorbol-12-myristate-13-acetate (PMA), as a positive control significantly decreased capillary numbers and increased capillary length after 24 h of exposition (Figure 4A). sEVs alone had no significant effect on both capillary number and length. As shown on Figure 4A–C, treatment with 40 µg/mL of BRB drastically reduced capillary number and length. Interestingly, the ability of sEV-loaded BRB (25 μg/mL) to reduce capillary networks (i.e., capillary number and length) was greater than free BRB.

### 3.6. Effects of Free BRB and sEV-Loaded BRB on NO Production

Finally, we examined endogenous NO production. As shown on Figure 4D,E, after 24 h of treatment, sEVs alone had no effect on NO levels, while free BRB (40 µg/mL) and sEV-loaded BRB (25 μg/mL) drastically decreased NO levels, sEV-loaded BRB being more potent than BRB alone.

## 4. Discussion

The present study shows that sEVs are potential stable BRB carriers with homogeneous particle size and steady zeta potential. More importantly, when loaded into sEVs, BRB is obviously more potent (up to 4-fold) than free BRB, regarding its ability to exert anti-tumor and antiangiogenic properties. These effects are associated with the increased inhibition of two major steps of tumorigenesis and angiogenesis, cell proliferation and migration. sEV-loaded BRB display anti-proliferative property accompanied by cell cycle arrest in G_1_ phase and cell migration in the two studied cell types. Interestingly, sEV-loaded BRB reduce the formation of capillary-like structures following the inhibition of endothelial NO release. These results show that sEVs may be considered as promising vehicles of BRB and as an innovative technology to target cancer and diseases characterized by an excess of angiogenesis.

One of the main technological challenges for the clinical employment of anticancer drugs is to establish a specific targeting of the bioactive molecules to their final cellular destination, to advance pharmacokinetics and long overdue of these drugs. Regarding BRB delivery, three types of delivery systems, i.e., solid lipid nanoparticles, nanoemulsions, and liposomes, have been described. Although the ratio of entrapment efficiency fluctuated between 78.5% to 97.6% [14,15] depending on the delivery system used, with nanoparticle sizes between 57 nm to 3.5 µm [14,16], all these delivery systems have major limitations after in vivo administration, mainly due to increased immunogenicity. For this reason, biophysical and biochemical properties of sEVs have been deeply studied to establish novel treatment horizons. Here, we have used sEVs derived from immature human dendritic cells to exclude immunogenicity [17]. To our knowledge, this is the first time that JAWS II-derived sEVs are used as a cargo of BRB. Taking into account the presented data, it seems that this nanobiomedicine approach is of interest since sEVs provided a combination of high EE, high drug loading, and good particle size. The EE of the formulation of sEVs loaded with BRB was 41.6%, implying that BRB was effectively loaded into the sEVs while the mean particle size determined by NTA of sEVs loaded with BRB was similar to native sEVs, indicating that the loading process did not affect the physical properties. These results are in accordance with curcumin in which the morphology and the size of sEVs after loading with curcumin were similar to native sEVs [18]. On the other hand, the low PDI value indicates a stable polydispersed suspension. In drug delivery applications using lipid-based carriers, such as liposome and nanoliposome formulations, a PDI of 0.3 and below is considered to be acceptable and indicates a homogenous population of phospholipid vesicles [19]. Besides, the zeta potential of sEVs loaded with BRB indicated that they exhibited a very good stability for loading BRB. These results demonstrate that sEVs are stable BRB carriers with homogeneous particle size and steady zeta potential. In line with these data, we have recently reported that sEVs from JAWS II could be good carriers to encapsulate the polyphenol delphinidin [13]. In fact, sEVs protect and probably limit delphinidin degradation in metabolites under the experimental conditions used. Other studies indicate that sEVs are stable vectors for paclitaxel delivery and they reported that the sEV-loaded paclitaxel (exoPTX) formulation is stable at various conditions for over a month, confirming previous reports about the long-term stability of sEVs [20]. In addition, sEVs may be lyophilized and reconstituted, while retaining their morphology and the activity of their cargo. This provides a clinical link for sEV-based drug formulations, suggesting that multiple lots of exoPTX may be prepared and stored prior to treatment. Nowadays, the potentiation of antiproliferative activity of BRB in various cancer cell lines has led to further research interest for this compound. Although we have not analyzed the profile of BRB release from sEVs, we have previously shown that the same type of sEVs is quickly internalized into cells [21]. In this study, we initially performed a proliferation test to determine the optimal concentration, at which BRB and sEV-loaded BRB inhibited cell proliferation. It was found that both free BRB and sEV-loaded BRB treatments inhibited cell proliferation in a time- and concentration-dependent manner. sEV-loaded BRB was 2 to 4 times more potent than free BRB on MDA-MB-231 and HUVECs in inhibiting proliferation, by a reduction of cell growth accompanied by cell cycle arrest at S phase, and prevented cell cycle progression to G2/M phase. It is well established that cell cycle progression is governed by a family of protein kinase complexes including the cki-cdk machinery. It has been described that such mechanisms can be involved for anti-cancer natural molecular compounds such as BRB [22]. In addition, migration of cells was decreased by treatments with free BRB and sEV-loaded BRB; these results being consistent with previous studies about anti-tumor activity of BRB on the MDA-MB-231 cell line [23]. Our results showed that BRB and sEV-loaded BRB could effectively affect HUVEC migration. We have reported using another polyphenol, delphinidin, that the inhibition of HUVEC migration is associated with a restoration of p27^kip1^, an important regulator of cell migration, and cyclin A expression [24]. Such mechanisms might underline the sEV-loaded BRB effects observed in our study. However, further studies are needed to understand which mechanism is controlling the observed effects.

In this study, free BRB and sEV-loaded BRB decreased capillary-like formation in an experimental model of angiogenesis essential for blood vessel formation in tumors. sEV-loaded BRB was twofold more potent than free BRB. A previous study described that the antiangiogenic activity of BRB is mainly mediated through the inhibition of various pro-inflammatory and pro-angiogenic factors including HIF, VEGF, COX-2, NO, and TNF-α [25]. We found that BRB decreased NO endothelial release and reduced angiogenesis [26], with a greater effect using sEV-loaded BRB. These results indicate that BRB loaded in sEVs are a promising approach to prevent pathologies associated with an excess of endothelial proliferation and vascularization, such as tumor development.

In summary, sEVs are potential carriers for BRB since they increased the efficacy of free BRB in inhibiting cell proliferation, cell migration, capillary-like formation, and NO release. Thus, sEVs represent a valuable delivery system with a good stability for BRB to treat cancer-related diseases by acting on both anti-tumoral and anti-angiogenic processes.

## Figures and Tables

**Table 1:** Entrapment efficiency, drug loading, mean particle size, polydispersity index (PDI), and zeta potential values of native sEVs and sEVs loaded with berberine (BRB).

	Entrapment Efficiency (%)	BRB Loading (µg of BRB/µg sEV)	Size (nm)	PDI	Zeta Potential (mV)
sEVs			101.13 ± 16.02	0.246 ± 0.01	−11.3 ± 8.4
sEV loaded with BRB	41.6 ± 1.8	3.57 ± 0.05	109.6 ± 11.5	0.327 ± 0.03	−5.4 ± 13 ****

Data are expressed as mean ± SEM of three independent experiments, **** *p* < 0.0001 sEVs loaded with BRB vs. sEVs.

**Table 2:** IC_50_ (µg/mL) values determined from cell proliferation assays for berberine (BRB) and BRB loaded into sEVs.

	MDA-MB-231		HUVECs	
	BRB	sEV-Loaded BRB	BRB	sEV-Loaded BRB
24 h	20 ± 0.12	5 ± 0.06 ****	40 ± 1.04	25 ± 0.76 ****
48 h	10 ± 0.15	2.5 ± 1.3 ****	25 ± 0.9	15 ± 0.3 ****
72 h	5 ± 0.21	1.25 ± 0.01 ****	15 ± 0.13	12 ± 0.22

Data are expressed as mean ± SEM of three independent experiments. IC_50_ expressed as µg/mL. **** *p* < 0.0001 vs. BRB.

## Data Availability

Almost all data are presented within the manuscript (figures and tables). The raw data presented in this study are available upon request to the corresponding author.

## Supplementary Materials

The following supporting information can be downloaded at: https://www.mdpi.com/article/10.3390/pharmaceutics14091913/s1, Figure S1: Quantification of cell cycle distribution on MDA-MB-231 (A) and HUVECs (B) after 24 h treatment with sEVs, BRB and sEV-loaded BRB. Data are expressed as mean ± SEM of three independent experiments. ** *p* < 0.01, **** *p* < 0.0001.

## Abbreviations

BRB	Berberine hydrochloride
%EE	entrapment efficiency
FBS	fetal bovine serum
HUVECs	human umbilical vein endothelial cells
NO	nitric oxide
NTA	nanoparticle tracking analysis
PMA	Phorbol-12-myristate-13-acetate
sEVs	small extracellular vesicles
TNBC	triple-negative breast cancer
