# Supplementary material for: Enhancement of the In Vitro Antitumor Effects of Berberine Chloride When Encapsulated within Small Extracellular Vesicles
Source: Pharmaceutics. 2022 Sep 9;14(9):1913. doi: 10.3390/pharmaceutics14091913 (PMC9500604; doi:10.3390/pharmaceutics14091913)
Supplement: Supplementary file 1 [file pharmaceutics-14-01913-s001.zip › pharmaceutics-1874345-supplementary.pdf]

A

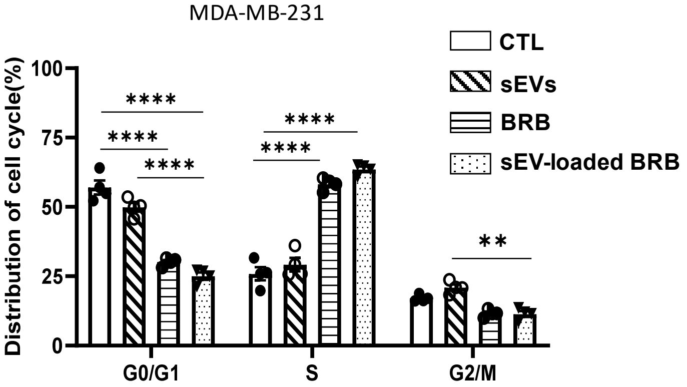

B

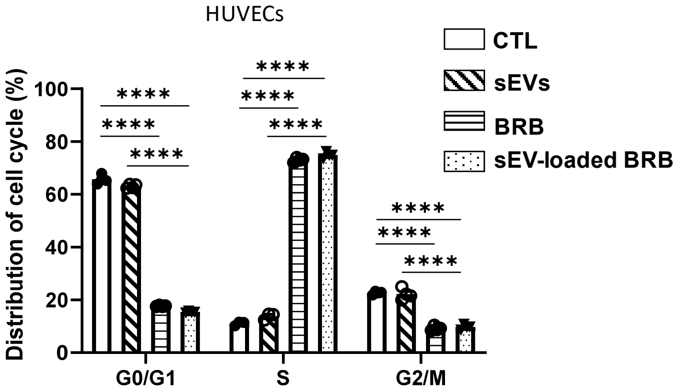

Supplemental Figure S1. Quantification of cell cycle distribution on MDA-MB-231 (A) and HUVECs (B) after 24 h treatment with sEVs, BRB and sEV-loaded BRB. Data are expressed as mean ± SEM of three independent experiments. ** $p < 0.01$, ****$p < 0.0001$.
